# Photo-conversion Methodology in Zebrafish Neural Mapping

**DOI:** 10.1093/geroni/igaf122.2265

**Published:** 2025-12-31

**Authors:** Alejandro Quezada, Adam Roberts

**Affiliations:** California State University, Fullerton, Santa Ana, California, United States; California State University, Fullerton, Fullerton, California, United States

## Abstract

Zebrafish possess unique attributes that make them ideal for brain imaging techniques, particularly those that enable in vivo tracking of neural activity. Their genetic modifiability, translucency, and ease of maintenance compared to other models make them well-suited for such research. Using a fluorescent protein called Kaede, real-time neural activity can be recorded by monitoring its fluorescence. Kaede fluorescence can be observed in two different states—green and red—through a process known as photo-conversion. Exposure to UV light induces an irreversible shift from green to red fluorescence, allowing researchers to track neural activity dynamically. This project aims to optimize the parameters involved in photo-conversion to improve efficiency. Enhancing these parameters will lead to better imaging quality and reduced phototoxicity, ultimately refining our ability to study neural circuitry and the molecular mechanisms underlying disordered behavior.

